# Acknowledgment of Reviewers, 2017

**DOI:** 10.1200/JGO.17.00088

**Published:** 2017-09-12

Peer review is at the core of scientific progress. It is only through careful critique and discussion that authors can add on to existing knowledge. The referee system also supports authors by making suggestions that can improve their work and give manuscripts validity and recognition.

As such, scientific outlets in general, and the Journal of Global Oncology in particular, as a new vehicle, owe an immense debt of gratitude to its Peer Reviewers.

In recognition of Peer Review Week 2017, we are pleased to recognize your efforts and thank you for your expertise and time by presenting this list of those who have contributed to our success.

For your hard work in 2017, our heartfelt THANK YOU!

Ghassan Abou-Alfa

Jame Abraham

Felipe Ades

Alvaro Alencar

Nada Alwan

Benjamin Anderson

Ramandeep Arora

Silvina Arrossi

Rashmi Asif

Fredrick Asirwa

Soe Aung

Baffour Awuah

Ronald Barr

Romualdo Barroso-Sousa

Michael Barton

Arnold Baskies

Partha Basu

Asim Belgaumi

Charles Bennett

P. Leif Bergsagel

Ines Beumer

Gouri Shankar Bhattacharyya

Stefan Bielack

Ines Buccimazza

Alfredo Carrato

Sauvaget Catherine

Amy Chadburn

Raymond Chan

Guillermo Chantada

Yanin Chavarri Guerra

Zvavahera Chirenje

Linus Chuang

James Cleary

Norm Coleman

Marcelo Cruz

Tanja Cufer

Maria Curado

Evandro de Azambuja

Paul de Souza

Egidio Del Fabbro

Jan Delabie

Lynette Denny

Vale Diama

Meletios Dimopoulos

Francois Doz

Jacqui Drope

Ira Dunkel

Allison Dvaldze

David Eisenstat

Ahmed Elzawawy

Alexandru Eniu

Beth Erickson

Adolfo Favaretto

Alberto Feletti

Gustavo Fernandes

Kenneth Fleming

Tamer Fouad

Silvia Franceschi

Maria Frech

David Gaffney

James Gajewski

Komal Galani

Prasanth Ganesan

Apar Kishor Ganti

Alberto Garaventa

Biju George

Anthony Gill

Jordi Giralt

Michael Goldstein

Satish Gopal

William Gradishar

KM Graner

Thomas Gross

Bishal Gyawali

Axel Hauschild

Mutlu Hayran

David Hill

Luciana Holtz C. Barros

David Hui

Andre Ilbawi

Thomas Inui

Farzad Jamshidi

Abdul-Rahman Jazieh

Robin Jones

Abhishek Joshi

Jean Marie Kabongo

Michael Kiehl

Eun Jeong Kim

Krishna Komanduri

Bridget Koontz

Geoffrey Ku

Lalit Kumar

Nilgün Kurucu

Donald Lannin

Pierre Laurent-Puig

MaryJo Lechowicz

Benjamin Levy

Chi Kong Li

Wan-Teck Lim

Jeffrey Lipton

Enriquito Lu

Cynthia Ma

Angelo Maiolino

Max Mano

Johanna Maree

Merry Markham

Sandro Martins

David Matchar

Aju Mathew

Danielle Matia

Mauricio Maza

Worta McCaskill-Stevens

Valerie McCormack

Cristina Meazza

Jane Mendez

Sofia Merajver

Monika Metzger

Danny Milner

Geoffrey Mitchell

Srabani Mittal

Anant Mohan

Alejandro Mohar

Toshikazu Moriwaki

Raul Murillo

Jonah Musa

Rajaram Nagarajan

Ambakumar Nandakumar

Ashrafun Nessa

Alfred Neugut

Lisa Newman

Twalib Ngoma

Anna Nowak

Emmanuel Oga

William Oh

Oluwatosin Omole

Ruth O'Regan

Groesbeck Parham

Purvish Parikh

Jong Sup Park

Simrit Parmar

Marc Peeters

Lester Peters

Usha Poli

Walter Prendiville

Holly Prigerson

Ibrahim Qaddoumi

Gwendolyn Quinn

Shalini Rajaram

Swaminathan Rajaraman

Evangelia Razis

Lorna Renner

Raul Ribeiro

Rachel Riechelmann

Danielle Rodin

Paul Rogers

Ju-Won Roh

Felipe Roitberg

Michelle Rowland

Paul Ruff

Raya Saab

Rodrigo Sanches Peres

Edgardo Santos

Shahin Sayed

Rebekkah Schear

Phillip Scheinberg

Eva Segelov

Saima Sharif

Surendra Shastri

Lawrence Shulman

Gerhard Sissolak

Lillian Siu

George Sledge

Enrique Soto-Perez-de-Celis

Dingle Spence

Virote Sriuranpong

Eva Steliarova-Foucher

Jaroslav Sterba

Raza Sughra

Richard Sullivan

Iyad Sultan

Perera Suraj

Mary Taj

Kimberson Tanco

Nelson Teich

Melissa Teo

Priya Tiwari

Cindy Tofthagen

Thuan Tran

Edward Trimble

George Tsermoulas

Gauri Varadhachary

Parthasarathy Vedasoundaram

RA Vicens

Claire Wagner

John Waldron

Harpreet Wasan

Béatrice Wiafe

Andres Wiernik

Yi-Long Wu

Kitsada Wudhikarn

Charlie Xue

Bilgehan Yalcin

Eddy Yang

Mei Ling Yap

Catheryn Yashar

Danny Youlden

Roberto Zanetti

Keary Zhou

Eduardo Zubizarreta

